# Prospective multi-center trial utilizing electronic brachytherapy for the treatment of endometrial cancer

**DOI:** 10.1186/1748-717X-5-67

**Published:** 2010-07-20

**Authors:** Adam Dickler, Mohamed Y Puthawala, John P Thropay, Ajay Bhatnagar, Gary Schreiber

**Affiliations:** 1 Little Company of Mary Hospital, Evergreen Park, IL USA; 2 Rhode Island Hospital, Providence, RI USA; 3 Beverly Oncology and Imaging Centers, Montebello, CA USA; 4 Cancer Treatment Services Arizona, Casa Grande, AZ USA; 5 Swedish Covenant Hospital, Chicago, IL USA

## Abstract

**Background:**

A modified form of high dose rate (HDR) brachytherapy has been developed called Axxent Electronic Brachytherapy (EBT). EBT uses a kilovolt X-ray source and does not require treatment in a shielded vault or a HDR afterloader unit. A multi-center clinical study was carried out to evaluate the success of treatment delivery, safety and toxicity of EBT in patients with endometrial cancer.

**Methods:**

A total of 15 patients with stage I or II endometrial cancer were enrolled at 5 sites. Patients were treated with vaginal EBT alone or in combination with external beam radiation.

**Results:**

The prescribed doses of EBT were successfully delivered in all 15 patients. From the first fraction through 3 months follow-up, there were 4 CTC Grade 1 adverse events and 2 CTC Grade II adverse events reported that were EBT related. The mild events reported were dysuria, vaginal dryness, mucosal atrophy, and rectal bleeding. The moderate treatment related adverse events included dysuria, and vaginal pain. No Grade III or IV adverse events were reported. The EBT system performed well and was associated with limited acute toxicities.

**Conclusions:**

EBT shows acute results similar to HDR brachytherapy. Additional research is needed to further assess the clinical efficacy and safety of EBT in the treatment of endometrial cancer.

## Introduction

Endometrial cancer is the most common gynecologic cancer, and an estimated 42,160 new cases of endometrial cancer were diagnosed in 2009 [1]. The standard management for endometrial cancer is a total abdominal hysterectomy and bilateral salpingo-oophorectomy (TAH-BSO) with or without lymph node sampling. The vagina is the most common site of recurrence, and whole pelvic radiotherapy, vaginal cuff brachytherapy, or both types of radiation therapy may follow surgical treatment. Radiation therapy significantly decreases the risk of local regional recurrence and has been associated with improved survival in patients with stage IC disease [2-4].

Vaginal brachytherapy is often employed in the treatment of endometrial cancer, either alone or in combination with external beam radiation. Vaginal brachytherapy has typically been delivered using a vaginal cylinder and a high dose rate (HDR) Iridium-192 radiation source. A modified form of HDR brachytherapy has been developed called Axxent Electronic Brachytherapy (EBT). The EBT device uses a 50 kilovoltage (kV) electronic X-ray source, which does not require a shielded vault for treatment or an HDR afterloader unit. The dosimetric properties of the EBT and Ir-192 sources were compared in the treatment of endometrial cancer [5]. Both sources provided equivalent target volume coverage, and EBT was associated with increased bladder and rectum sparing compared to Ir-192.

A prospective, multi-center clinical study was carried out to evaluate the success of treatment delivery, safety and toxicity of EBT as post-surgical adjuvant radiation therapy in patients with early-stage endometrial cancer. The results of this trial represent the first clinical report of EBT in the treatment of endometrial cancer.

## Methods

The study was approved at the institutional review boards at each of the five participating sites. Each patient was consented prior to enrollment in the trial.

### Patients

This trial utilized the FIGO staging system for endometrial cancer developed in 1988. Eligibility for the trial included patients with Stage I & II endometrial cancer, excluding Stage IA Grade 1, who had undergone a TAH-BSO. Exclusion criteria included patients with collagen vascular disease, scleroderma, or active lupus.

### Materials

The EBT system consists of the disposable X-ray source, vaginal applicators, the controller unit, and the base plate and clamp. The miniature X-ray source produces 50 kilovolt X-rays at its tip and can be translated within the applicator to provide a predictable dose of radiation to the tissue surrounding the cylinder. The vaginal applicators are cylinders made of medical-grade polymers and provide transmission characteristics specifically for the low energy X-rays emitted by the EBT source. A vaginal cylinder size was selected for each patient, and 25 mm, 30 mm, 35 mm cylinders were utilized in the study. The applicator was inserted just prior to treatment and removed following treatment on each treatment visit. The base plate and clamp provide stabilization of the applicator during radiation treatment. The mobile controller unit provides power to the X-ray source and contains the user interface.

### Treatment

If vaginal brachytherapy was to be administered as the sole radiation treatment modality, sites were given the option of treating with a prescription dose 7.0 Gy x 3 to 0.5-cm depth or 5.5 Gy x 4 to 0.5-cm depth. If vaginal brachytherapy was to be delivered in conjunction with external beam radiation therapy (EBRT), sites first delivered 45 Gy EBRT in 25 fractions to the pelvis. At the completion of EBRT, sites were given the option of treating with an EBT prescription dose of 6.0 Gy x 3 to the vaginal surface or 8.0 Gy x 2 to the vaginal surface.

Treatment planning was performed according to the standard of care at the treating institution and typically with BrachyVision™ treatment planning software (Varian Medical Systems, Palo Alto, CA) or Plato™ treatment planning software (Nucletron, Columbia, MD).Three dimensional treatment planning was completed for each patient prior to the first brachytherapy fraction. Both 2D and 3D treatment planning were permitted prior to each fraction according to the physician's standard of care, but a 3D treatment plan based on computed tomography (CT) was required so that normal tissue doses could be calculated. CT images were recorded prior to each fraction on all patients to verify correct applicator placement. A CT scan was performed with the vaginal applicator in place and the patient in a supine position. The CT scan encompassed a superior border of L5/S1 and an inferior border of the ischial tuberosities. TG-43 parameters specific to EBT were used to compute the delivered dose [5,6]. Patients were followed at 1 month and 3 months post-treatment.

### Endpoints

The primary endpoints of the study were the successful delivery of the prescribed radiation dose and treatment-related adverse events. Adverse events and severity were recorded during treatment and at the 1- and 3-month follow-up visits. Adverse events were graded according to the common terminology criteria (CTC) version 3.0. The n (number of observations) and proportion is reported for each endpoint. For continuous variables, the mean, standard deviation, and range is presented. Categorical variables are described using proportions and frequencies.

## Results

### Patient Population

A total of 15 patients were enrolled in the study. The first patient was enrolled in September 2008, and enrollment was completed in October 2009. Patient and disease characteristics are listed in Table 1. The mean age of the patients was 63.2 years of age (range 41.6-72.7). Nearly half (46.7%) had FIGO Stage IB cancer; 5 (33.3%) had Stage IC, and 3 (20.0%) had Stage IIA. All patients were followed for 3 months.

**Table 1:** Patient Characteristics

Mean Age (Range) Years	63.2 (41.6-72.7)
**Race**	**n (%)**

Caucasian	11 (73.3%)
Hispanic	1 (6.7%)
Asian	2 (13.3%)
Other	1 (6.7%)
**FIGO Cancer Stage**	
IB	7 (46.7%)
IC	5 (33.3%)
IIA	3 (20.0%)
**Tumor Grade**	
Grade 1	3 (20.0%)
Grade 2	8 (53.3%)
Grade 3	4 (26.7%)
**Depth of Myometrial Invasion**	
≤1/3	8 (53.3%)
> 1/3 and ≤ 2/3	5 (33.3%)
> 2/3	2 (13.3%)
	

**Mean Time from Hysterectomy to 1st EBT Treatment**	113.1 Days
**(Range)**	(37-787) Days

**Applicator Sizes**	**n (%)**
25 mm	7 (46.7%)
30 mm	7 (46.7%)
35 mm	1 (6.7%)

### Treatment

The EBT vaginal brachytherapy was successfully delivered for all 48 treatments in the 15 patients. In the 10 patients who received EBT alone, the prescription dose was 7.0 Gy x 3 fractions to a 0.5 cm depth in 7 patients and 5.5 Gy x 4 fractions to a 0.5 cm depth in 3 patients (Table 2). In the 5 patients who received EBRT before EBT, the EBRT dose was 45 Gy in 25 fractions in 4 patients delivered by IMRT in 3 and three dimensional conformal radiation therapy (3D-CRT) in 1, and 50.4 Gy in 28 fractions in 1 patient delivered by 3D-CRT. Following EBRT, the EBT prescription dose was 6.0 Gy x 3 fractions to the vaginal surface in 3 patients, 5.0 Gy x 4 to the vaginal surface in 1 patient, and 8.0 Gy x 2 to the vaginal surface in 1 patient. The mean treatment time was 4.9 minutes. The brachytherapy treatment summary and applicator size used for each patient is listed in Table 1. CT scans were used to evaluate the dose to normal tissues and volume of treatment after applicator insertion and prior to the first fraction of brachytherapy. The length of vagina treated ranged from 4.0 to 7.0 cm with a mean length of 5.28 cm. The dosimetric data is summarized in Table 3.

**Table 2:** Total Prescribed Dose (Gy) of EBT in patients categorized by whether they received EBRT in addition to EBT

	EBT Alone		EBT + EBRT Prescription		
Dose (Gy)	5.5Gy x 4Fx to 0.5 cm	7Gy x 3Fx to 0.5 cm	8Gy x 2Fx to Vaginal Surface	6Gy x 3Fx to Vaginal Surface	5Gy x 4Fx to 0.5 cm

# of patients (%)	3 (20.0%)	7 (46.7%)	1 (6.7%)	3 (20.0%)	1 (6.7%)

Gy = gray, EBRT= external beam radiation therapy, Fx = Fraction, cm = centimeter

**Table 3:** Dosimetry Analysis: The percent of the planned target volume (PTV) or organ receiving 50, 95, 100, or 150% of the prescribed dose at depth followed by the maximum point dose in cGy to the indicated organ

	Mean % ± SD	Range
%V95	91.0 ± 13.6	49.0 - 103.0
%V100	87.6 ± 13.7	48.0 - 98.0
%V150	34.1 ± 15.6	3.3 - 69.7
Bladder %V50	11.5 ± 9.7	0 - 40.2
Rectal %V50	17.4 ± 10.9	0 - 36.0
		

Max Point Dose to Bladder	701.2 ± 169.3 cGy	467 - 1087 cGy
Max Point Dose to Rectum	775.0 ± 355.4 cGy	100 - 1584 cGy
Max Point Dose to Small Bowel	421.3 ± 391.1 cGy	0 - 1188 cGy

The EBT system performed without major malfunction. No technical issues occurred with the controller or the applicators. At one site there was a source issue related to the electrical connection, which was traced to a loose clamp assembly. This issue was easily rectified, and treatment was completed as scheduled.

### Adverse Events

An independent data safety monitor adjudicated the adverse events. Six patients reported adverse events possibly or probably related to the EBT treatment including 4 CTC Grade I toxicities and 2 CTC Grade II toxicities (Table 4). There were no treatment related adverse events reported at the time of treatment and there were no serious adverse events reported in the study. One patient developed Grade I dysuria at her 1-month follow-up visit. Additional Grade I adverse events reported by one patient each included mild mucosal atrophy, vaginal drying, and rectal bleeding. A patient reported both Grade II dysuria and pelvic pain at her 1-month follow-up visit. Nine of 15 patients reported no treatment related adverse events during treatment through the 3-month follow-up visits.

**Table 4:** Number (%) of patients with adverse events reported at the one-month (1 mo) or three-month (3 mo) follow-up visit that are possibly related or probably related to the EBT treatment

1 Month Visit							
**Pt #**	**RT**	**Adverse Event**	**Grade**	**N (%)**	**Visit**	**Relationship to EBT Treatment**	**Visit Resolved**

A	EBT & EBRT	Dysuria	1	1	1 mo	Possibly related	Unresolved at 3 mo. visit
B	EBT & EBRT	Dysuria	2	1	1 mo	Possibly related	Resolved at 3 mo. visit
C	EBT & EBRT	Vaginal pain	2	1	1 mo	Probably related	Resolved at 6 wk. visit

**3 Month Visit**							

**Pt #**	**RT**	**Adverse Event**	**Grade**	**N (%)**	**Visit**	**Relationship to EBT Treatment**	**Visit Resolved**

D	EBT	Mucosal atrophy	1	1	3 mo	Probably Related	Reported at 3 mo. visit
E	EBT	Rectal bleeding	1	1	3 mo	Probably Related	Reported at 3 mo. visit
F	EBT & EBRT	Vaginal Drying	1	1	3 mo	Possibly Related	Reported at 3 mo. visit

EBT = Electronic Brachytherapy, EBRT = External Beam Radiation Therapy RT = radiation therapy treatment, mo = month, wk = week

## Discussion

Post-operative vaginal brachytherapy was compared with external beam radiation therapy (EBRT) in 427 patients with stage I or IIA endometrial cancer in a report of the PORTEC-2 trial [7]. The rates of overall survival, disease free survival, and vaginal relapse were not significantly different between the two treatment modalities. However, the rates of Grade I-II gastrointestinal toxicity were significantly lower in the vaginal brachytherapy arm (27/215 patients or 12.6%) as compared with the EBRT arm (112/208 patients or 53.8%). The authors of this study concluded that vaginal brachytherapy alone should be the adjuvant treatment of choice for patients with high-intermediate risk endometrial cancer [7]. Those results may lead to more patients being treated with post-surgical vaginal brachytherapy alone for early stage endometrial cancer. This combined with patients who receive both EBRT and vaginal brachytherapy likely will lead to an increasing utilization of HDR vaginal brachytherapy.

Currently, the most common method of delivering vaginal brachytherapy relies on a radioactive isotope, Iridium-192, which is not feasible for all centers. Many centers do not have an HDR afterloader device, which is required with an Ir-192 source. In addition, many centers have a single shielded radiation vault for both their EBRT and HDR patients. This can lead to logistical difficulties in scheduling patients at a busy radiation center. Electronic brachytherapy (EBT) was developed to make brachytherapy more accessible for patients. EBT treatment does not require a shielded radiation bunker and thus increases the settings in which brachytherapy treatments can be performed.

This report describes the first prospective clinical trial of vaginal EBT for the treatment of endometrial cancer. EBT treatment was delivered successfully for all 48 fractions of treatment in this study. The EBT device performed as expected with minimal technical issues. EBT was well tolerated with no serious adverse events. Six patients reported Grade 1-2 adverse events. Previous reports of EBT for accelerated partial breast irradiation (APBI) demonstrated an acceptable safety profile similar to that seen with Ir-192 based APBI [8]. Previous reports with Ir-192 based vaginal brachytherapy have shown it to be a very well tolerated procedure. Fayed, et al., reported only a 4% risk of Grade III/IV toxicity in 175 patients treated with HDR. The authors also noticed that the complication risk was higher if the patients also received EBRT [9]. Weiss et al and MacLoed et al have both reported on studies with over 100 patients treated with HDR brachytherapy alone and described no Grade III/IV toxicity [10,11]. It should be noted that these studies utilizing Ir-192 have larger patient numbers and longer follow-up than the current series.

The EBT radiation fractionation schedules utilized in this study were derived from the American Brachytherapy Society Recommendations for the suggested doses of Ir-192 HDR alone or in combination with EBRT [12]. It has previously been shown by Dickler, et al., in a dosimetric comparison that EBT offers similar target volume coverage and increased bladder and rectum sparing compared to Ir-192 based vaginal brachytherapy [5]. As a result, using the same radiation fractionation as used for Ir-192 treatment, it is reasonable to expect similar or possibly less bladder and rectal toxicity with EBT treatment. In the current study at 3 months follow-up, there have been no reports of Grade III/IV toxicity, and 9 of 15 patients have reported no toxicity at all. This is consistent with previous published reports using Ir-192 brachytherapy [7,10,11].

Although the study by Dickler, et al., showed similar target coverage between EBT and Ir-192 HDR treatment, EBT was associated with increased "hot spots" in the vaginal canal [5]. Specifically, the %V150 (percent of the target volume receiving 150% of the prescription dose) was 58.9% vs. 35.8% for the EBT and Ir-192 treatments, respectively. It is not known whether an increased volume of the vaginal canal being exposed to higher radiation doses will put patients at an increased risk for vaginal side effects such as stenosis or vaginal shortening. At 3 months follow-up, there were no incidences of vaginal stenosis or shortening in the current study. Of note, Noyes and investigators from University of Wisconsin have reported their results treating 63 patients with HDR and vaginal ovoids with vaginal surface doses of 16.2 Gy. The authors reported no incidence of Grade III/IV side effects using much higher vaginal surface doses than used in the current study [13]. Further follow-up will be needed to determine if late vaginal side effects occur at an increased rate with EBT treatment.

## Conclusions

The EBT system performed well and was associated with limited acute adverse events. The prescribed dose was successfully delivered in all 15 patients. Acute results are similar to those using HDR brachytherapy. Further research with EBT will be needed to establish its clinical efficacy and long-term toxicity in the treatment of patients with endometrial cancer.

## List of Abbreviations

AE: Adverse events; CT: Computerized tomography; CTC: Common Terminology Criteria; EBRT: External Beam Radiation Therapy; EBT: Electronic Brachytherapy; Gy: Gray; HDR: High Dose Rate; Ir- 192: Iridium 192; kV: kilovoltage; QID: Four times per day, TAH-BSO: total abdominal hysterectomy and bilateral salpingo-oophorectomy

## Competing interests

We disclose to Radiation Oncology the following potential conflicts of interest:

Author Disclosures: Dr. Dickler is on the scientific advisory board for Xoft, Inc.

## Authors' contributions

All five authors contributed significantly to this manuscript by contributing to the study data collection, reviewing the data analyses, revising, and approving the final manuscript. All authors contributed to the study design of this first experience.

## Author's Information

AD has completed dosimetric comparisons of electronic brachytherapy and iridium-192 and requested to proceed with a small study on the initial experience using electronic brachytherapy for the treatment endometrial cancer following TAH-BSO.
